# Feasible and acceptable social drivers of health screening among patients with chronic liver disease

**DOI:** 10.1097/HC9.0000000000000758

**Published:** 2025-06-30

**Authors:** Rebecca G. Kim, April Ballantyne, Rachel R. Codden, Morgan M. Millar, Andrea Wallace, John M. Inadomi, Molly B. Conroy, Jennifer C. Price

**Affiliations:** 1 Department of Internal Medicine, Division of Gastroenterology and Hepatology, University of Utah School of Medicine, Salt Lake City, Utah, USA; 2 Department of Internal Medicine, Division of Epidemiology, University of Utah School of Medicine, Salt Lake City, Utah, USA; 3 College of Nursing, University of Utah, Salt Lake City, Utah, USA; 4 Department of Internal Medicine, Division of General Internal Medicine, University of Utah School of Medicine, Salt Lake City, Utah, USA; 5 Department of Medicine, Division of Gastroenterology and Hepatology, University of California, San Francisco, California, USA

**Keywords:** food insecurity, health disparities, social needs

## Abstract

**Background:**

Social drivers of health (SDoH) contribute to health disparities among patients with chronic liver disease (CLD). Little is known about the feasibility and acceptability of SDoH screening in hepatology clinics. This study aimed to define SDoH prevalence among CLD patients, identify a feasible and acceptable screening approach, and assess the convergent validity of a locally developed screener.

**Methods:**

Among adult patients with CLD receiving care in hepatology clinics, 2 SDoH screeners were administered to eligible participants: (1) default electronic medical record (EMR) questions and (2) Screener for Intensifying Community Referrals for Health (SINCERE). The primary outcomes were (1) prevalence of SDoH, (2) SDoH screening feasibility and acceptability, and (3) factors associated with screening acceptability. As a secondary outcome, the convergent validity of SINCERE to EMR was assessed.

**Results:**

Among 250 participants, the mean age was 56 years, 56% were women, 22% were Hispanic, 7% were American Indian/Alaska Native, 58% had cirrhosis, 29% completed high school or less, 22% were unemployed/disabled, and 29% had an annual income <$35,000. Based on SINCERE, 26% had food insecurity, 8% transportation needs, 43% financial strain, 5% lack of social support, and 24% housing instability. Most respondents (69%) were comfortable or very comfortable completing SDoH screening. Using the McNemar test, there were statistically significant differences between screeners for financial strain and housing instability.

**Conclusions:**

Among CLD patients at our center, SDoH were prevalent, and screening within the hepatology clinic was feasible and acceptable. To detect social needs, SINCERE, a locally developed screener, had overall acceptable convergent validity. These data support SDoH screening in hepatology clinics. Future multicenter studies evaluating the effective implementation of SDoH screening for CLD patients, including contextualized care plans and connection to available resources, should be conducted.

## Graphical abstract

**Figure:**
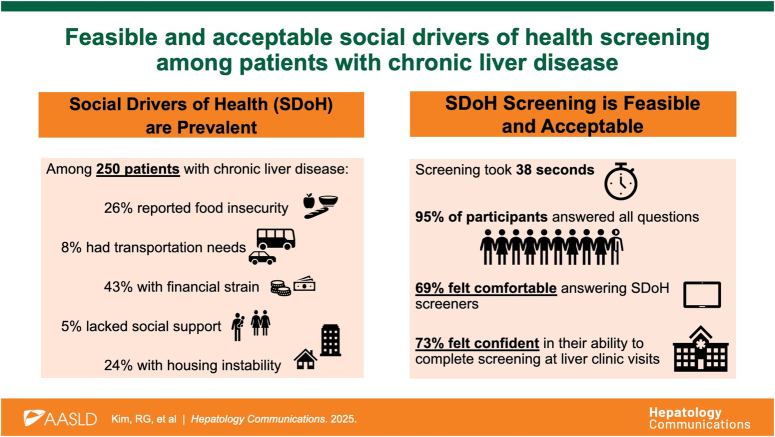

## INTRODUCTION

Social drivers of health (SDoH, also referred to as social determinants of health),^1^ the conditions where people are born, live, work, and age, lead to avoidable health differences experienced by socially disadvantaged populations (ie, health disparities).^2^,^3^,^4^ Populations, including low-income individuals and racial and ethnic minority groups, are more negatively affected by SDoH like food insecurity (FI), housing instability, stress, and prejudice, resulting in worse chronic disease outcomes and increased emergency department utilization and hospitalizations.^5^,^6^,^7^,^8^ Chronic liver disease (CLD), affecting 4.5 million adults in the United States, is directly impacted by SDoH, and vulnerable populations experience a higher incidence of CLD and CLD mortality.^3^,^9^,^10^,^11^,^12^

CLD, including cirrhosis, was the ninth leading cause of death in the United States in 2021, but the eighth and fifth leading cause of death among Hispanic and American Indian/Alaska Native (AI/AN) populations, respectively.^9^ There is an increased incidence of cirrhosis among people without health insurance.^13^ Liver cancer is often diagnosed in later stages in Hispanic and Black patients, vastly limiting treatment options and survival.^12^,^14^ Racial and ethnic minority groups are also less likely to be listed for liver transplant or receive living donor liver transplants.^3^,^15^ Moreover, differences are seen in CLD risk factors such as alcohol use, obesity, diabetes, and hyperlipidemia.^3^,^7^,^16^

**FIGURE 1:**
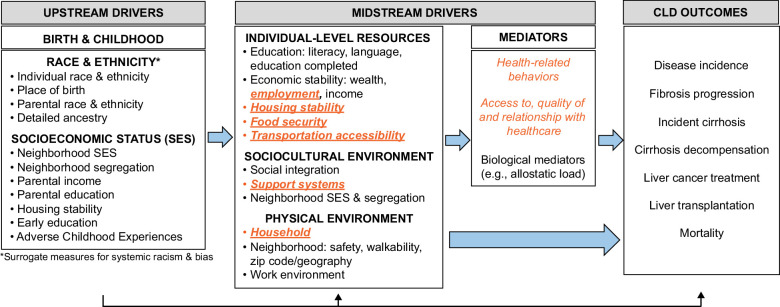
Conceptual framework for social drivers of health (SDoH), their proposed interactions and relationship on chronic liver disease outcomes. *Orange factors* are potentially modifiable SDoH at the clinic or health system level, and factors bolded and underlined are health-related social needs screened for in the proposed SDoH screeners.

Importantly, some SDoH, often referred to as health-related social needs (HRSNs, Figure 1), like FI, are potentially modifiable at the clinic or health system level with the use of community resources.^6^ In fact, blood pressure, cholesterol levels, hypoglycemia, and depressive symptoms improved when patients were screened for HRSN and connected with resources.^17^,^18^,^19^ To effectively intervene on SDoH to mitigate their effect on CLD, they must first be identified through adequate screening methods. SDoH screening in hepatology clinics provides an opportunity for identification and intervention on SDoH.

Standardized SDoH or HRSN screening has not been defined,^20^ and only a small number of studies have specifically assessed screening among patients with CLD.^16^,^21^,^22^ Although SDoH screening has previously been shown to be acceptable,^20^,^23^ most studies were conducted in primary care or pediatrics, where familiarity with the clinic staff or provider may differ from hepatology clinic. These relationships, along with patients’ expectations of the visit, may potentially affect patients’ comfort completing SDoH screeners.^24^ Therefore, this study aimed to (1) define the prevalence of SDoH among patients with CLD seen in hepatology clinics, (2) assess SDoH screening feasibility and acceptability, and (3) identify factors associated with screening acceptability. Lastly, because the SDoH screener used at our center is a locally developed screener focused on identifying HRSN, we aimed to assess its convergent validity via comparison to the default electronic medical record (EMR) SDoH screener.

## METHODS

### Conceptual framework

Figure 1 depicts a relationship between SDoH, categorized as upstream and midstream drivers, and their impact on CLD-related outcomes.^25^,^26^,^27^,^28^ In this framework, SDoH are categorized into early causal factors (upstream drivers), potentially modifiable factors (midstream drivers), and downstream effects (CLD outcomes). Upstream drivers are societal factors contributing to the complex relationship between race, ethnicity, and socioeconomic status that define social status early in life. Within this framework, upstream drivers influence midstream drivers, and both have a causal relationship with poor health outcomes. Behavioral factors, biological mediators, and healthcare access can be considered midstream drivers; they uniquely act as mediators contributing their effect through an additional causal pathway. The orange drivers are potentially modifiable midstream drivers, or HRSN, specifically at the clinic or healthcare system level. They can serve as potential targets of healthcare-based interventions.

### Study cohort

From September 2023 to February 2024, 250 adult patients (ages ≥18) with CLD were recruited from hepatology clinics at the University of Utah Health (UUH). The EMR of all patients scheduled in UUH hepatology clinics was screened by study investigators prior to clinic visits. Patients with a confirmed diagnosis of any etiology of CLD [ie, metabolic dysfunction–associated steatotic liver disease (MASLD), alcohol-associated, viral, autoimmune/cholestatic, Wilson disease, hereditary hemochromatosis, Budd–Chiari, alpha-1 antitrypsin, cirrhosis) were included. Patients with cognitive impairment or psychiatric conditions affecting their ability to consent and non-English or Spanish-speaking patients were excluded. All research in this study was conducted in accordance with both the Declarations of Helsinki and Istanbul, and all research was approved by the Institutional Review Board of the University of Utah.

### Screener selection

There are many SDoH screeners available for use, and there is no formal recommendation for the use of one over another. When selecting a screener, it is important to define the goal of screening for the hospital system and patient population. Nationally, one of the most used EMR systems (Epic Systems) has included an SDoH screener within their system. It is a 17-question screener that asks about alcohol use, physical activity, as well as social risks.^29^,^30^,^31^,^32^,^33^,^34^,^35^,^36^,^37^ Due to its use of validated, widely published questions and broad availability for use, we selected the EMR screener as our gold standard.

At our institution, SDoH screening has been conducted in various clinical settings by co-author Dr. Andrea Wallace and her team.^38^,^39^ Their screening has used SINCERE, the Screener for Intensifying Community Referrals for Health. SINCERE was developed in 2017 when a workgroup of academic and community service partners reviewed assessment tools from payors and nonprofit organizations to identify key principles for social needs assessment in emergency departments, focusing on actionable information, clear communication principles, and a consistent response scale. They adapted and refined questions from existing tools to ensure relevance, consistency across English and Spanish translations, and avoidance of duplication. The screening questions were initially tested through a 2-phase, mixed-methods feasibility study, have been psychometrically validated, and have been incorporated in additional studies.^39^

For the health system’s and community partners’ priorities, SINCERE was proposed to become the default SDoH screener, and was integrated into the medical record system. From a research perspective, before expanding the use of SINCERE to the entire healthcare system including outpatient subspecialty clinics, we aimed to assess its convergent validity among our patient population.

### Survey administration

The SDoH screening questions were entered into REDCap^40^,^41^ along with questions about patient demographics and additional SDoH not otherwise captured, including age, race, ethnicity, gender, sexual orientation, place of birth, primary and preferred languages, and geographic location (Figure 1). Lastly, participants were asked questions assessing the acceptability of SDoH screening, which were adapted from prior studies, one with an overall acceptability score.^42^,^43^ The survey and consent forms were translated into Spanish.

Patients were approached prior to or after their hepatology visit in the outpatient clinic room, where the purpose and details of the study were explained. An electronic consent form was provided to read and sign if patients expressed interest. As compensation for time, each participant received $10.

The complete survey was administered through an iPad. The survey was 45 questions with up to 3 additional supplemental questions depending on responses. The SINCERE and EMR screeners were answered by all participants; however, the order in which the screeners were administered (ie, SINCERE then EMR or EMR then SINCERE) was randomly assigned. All participants were provided the survey to complete on their own, including a certified Spanish version, via REDCap. Although everyone was encouraged to complete the survey independently, a research team member and a Spanish interpreter, as needed, were available for questions. Survey completion took ~10–15 minutes, longer if a Spanish interpreter was present. The survey was read verbatim if requested by the patient. All patients were provided information on community resources.

### Measures

Both SDoH screeners, EMR and SINCERE, were used to assess for (1) FI, (2) transportation needs, (3) financial strain, and (4) lack of social support. Housing instability was also assessed by SINCERE; however, not by EMR. Therefore, housing instability questions from PhenX,^44^ a web-based catalog of recommended measurements for use in research with human participants, were used for comparison. The screeners and definitions of each SDoH are included in Supplemental Table S1, http://links.lww.com/HC9/C42.

The following demographic characteristics and SDoH were self-reported: gender identity, race, ethnicity, sexual orientation, education, employment, annual income, health insurance, place of birth, primary language, and preferred language. Zip codes provided by participants were used for geographic categorization (eg, urban, rural) according to the U.S. Department of Agriculture Rural–Urban Commuting Area Codes.^45^,^46^

Feasibility was assessed using study participation rate, time required to complete each screener (in seconds), missingness of questions from each screener, and the proportion of screeners completed in their entirety.

Acceptability was assessed using 7 items: affective attitude, ethicality, coherence, perceived effectiveness, self-efficacy, burden, and opportunity costs. A composite acceptability score was summed.^42^ Reverse coding was done for the burden and opportunity costs items. The total acceptability score for each participant could range from 7 to 35, with higher values indicating higher acceptability.

### Outcome measures

The primary outcomes were (1) prevalence of SDoH among CLD patients, (2) the feasibility and acceptability of SDoH screening in UUH hepatology clinics, and (3) factors associated with screening acceptability. The secondary outcome was the convergent validity of SINCERE compared to the EMR screener for detecting SDoH.

### Statistical analysis

Descriptive statistics were calculated (counts and percentages for categorical data, and median and IQR or mean and SD for continuous measures), as well as prevalence of SDoH (proportion and 95% CI). Fisher exact, Monte Carlo approximation of Fisher exact, Kruskal–Wallis and Wilcoxon rank sum tests were used to test for association between demographic variables and SDoH and feasibility and acceptability measures.

To assess the convergent validity of SINCERE, the 2 screeners’ classifications of FI, transportation needs, financial strain, social support, and housing instability were used to calculate sensitivity, specificity, positive predictive value, negative predictive value, a measure of agreement (Cohen kappa), and a test for bias (McNemar). For purposes of these analyses, the EMR screener was considered the gold standard for comparison.

Analyses were completed with SAS 9.4. *p* values <0.05 are considered statistically significant.

## RESULTS

### Study participants

Of the 250 participants, the mean age was 56 years, 56% were women, 67% were White, 22% were Hispanic or Latino/a/a, 3% were Asian, 7% AI/AN, 63% had MASLD, and 58% had cirrhosis. Ten participants completed the survey in Spanish. Nearly one-third (29%) completed a high school education or less, 22% were unemployed or disabled, and 29% reported an annual income of <$35,000 (Table 1).

**TABLE 1:** Demographic characteristics of adults with chronic liver disease in the study population, N=250^a^

Characteristic	Participants, N (%)
Age, mean (SD), y	55.7 (14.3)
Gender identity (N=247)	
Man	109 (44.1)
Woman	137 (55.5)
Non-binary	1 (0.4)
Race or ethnicity	
American Indian or Alaska Native	18 (7.3)
Asian	8 (3.2)
Black or African American	8 (3.2)
Hispanic or Latino/a	54 (21.8)
Native Hawaiian or Pacific Islander	3 (1.2)
White	167 (67.3)
Sexual orientation (N=240)	
Gay	6 (2.5)
Straight	213 (88.8)
Bisexual	2 (0.8)
Other	4 (1.6)
Prefer not to answer	15 (0.6)
Highest level of school completed (N=247)	
Less than a high school graduate/GED	72 (29.2)
2-year college degree or some college, no degree	100 (40.5)
4-year college degree or more	75 (30.4)
Current occupational status	
Employed	101 (40.2)
Unemployed	30 (12.3)
Homemaker/Student	20 (8.1)
Disabled/Retired	93 (37.1)
Other	6 (2.5)
Combined annual income (N=246)	
$0–$34,999	63 (25.6)
$35,000–$99,999	83 (33.7)
>$100,000	71 (28.9)
Don’t know/prefer not to answer	29 (11.8)
Birthplace (N=232)	
United States	195 (84.1)
Geographic classification of primary residence (N=247)	
Urban	194 (78.5)
Large rural city/town	31 (12.6)
Small and isolated small rural town	22 (8.9)
Geographic classification by state	
Utah	195 (78.9)
Urban	178 (91.3)
Large rural city/town	11 (5.6)
Small and isolated rural town	6 (3.1)
Idaho	20 (8.1)
Urban	11 (55.0)
Large rural city/town	5 (25.0)
Small and isolated small rural town	4 (20.0)
Wyoming	11 (4.5)
Large rural city/town	7 (63.6)
Small and isolated small rural town	4 (36.4)
Nevada	10 (4.0)
Large rural city/town	5 (50.0)
Small and isolated small rural town	5 (50.0)
Montana	6 (2.4)
Urban	1 (16.7)
Large rural city/town	3 (50.0)
Small and isolated small rural town	2 (33.3)
Other	5 (2.0)
Urban	4 (80.0)
Small and isolated small rural town	1 (20.0)
First language	
English	207 (82.8)
Spanish	28 (11.2)
Other	15 (6.0)
Language preference with healthcare team (N=249)	
English	236 (94.8)
Spanish	11 (4.4)
Other	2 (0.8)
Etiology of liver disease	
Cirrhosis and complications	145 (58.0)
MASLD	158 (63.2)
Alcohol-associated	70 (28.0)
Viral hepatitis	48 (19.2)
Autoimmune/cholestatic	50 (20.0)
Other	5 (2.0)
Physical activity (N=216)^b^	
Patients who self-reported any physical activity	169 (69.5)
Patients who self-reported no physical activity	74 (30.5)
Alcohol use^b^	
How often do you have a drink containing alcohol? (N=248)	
Never	203 (81.9)
Monthly or less	22 (8.9)
2–4 times per month	9 (3.6)
2–3 times per week	5 (2.0)
4 or more times per week	9 (3.6)
How many drinks containing alcohol do you have on a typical day when you are drinking? (if ever drink) (N=44)	
1 or 2	32 (72.7)
3 or 4	7 (15.9)
5 or 6	1 (2.3)
7–9	4 (9.1)
10 or more	0 (0.0)
How often do you have 6 or more drinks on one occasion? (if ever drink) (N=45)	
Never	25 (55.6)
Less than monthly	12 (26.7)
Monthly	2 (4.4)
Weekly	4 (8.9)
Daily or almost daily	2 (4.4)

^a^ Unless otherwise defined in the table.

^b^ Questions included in the Electronic Medical Record social drivers of health screener.

Abbreviations: GED, General Educational Development; MASLD, metabolic dysfunction–associated steatotic liver disease.

### Prevalence of modifiable SDoH or health-related social needs

Based on SINCERE, SDoH (proportion, 95% CI) were reported as the following: 26% (20.4–31.7) with FI, 8% (4.4–11.1) transportation needs, 43% (36.5–48.9) financial strain, 5% (2.0–7.6) lack of social support, and 24% (18.5–29.9) housing instability (Table 2). Most values were similar when using the EMR screener (Table 2): 30% (24.4–36.1) with FI, 9% (5.7–13.1) transportation needs, and 4% (1.4–6.5) lack of social support. However, only 8% (4.7–11.5) reported financial strain using the EMR screener (vs. 43% with SINCERE). Lastly, 48% (40.9–54.1) reported housing instability according to the PhenX housing screener.

**TABLE 2:** Responses to social drivers of health screening. (A) Screener for intensifying community referrals for health (SINCERE) and (B) electronic medical record (EMR) screener, N=250^a^

(A) SINCERE	N (%)	(B) EMR screener	N (%)
In the past year…			
Housing instability			
Was there a time when you were not able to pay your mortgage or rent? (N = 248)		^a^PhenX questions were used for comparison	
Yes	50 (20.2)		
Was there a time when you were not able to pay your utility bills? (N=247)			
Yes	51 (20.6)		
Have you slept outside, in a shelter, in a car, or in any place not meant for sleeping? (N=249)			
Yes	12 (4.8)		
Food insecurity			
Did you feel there was not enough money for food? (N=249)		Within the past 12 months, you worried that your food would run out before you got money to buy more (N=248)	
Yes	67 (26.9)	Never true	178 (71.8)
		Sometimes true	55 (22.2)
		Often true	15 (6.0)
		Within the past 12 months, the food you bought just didn’t last and you didn’t have money to get more? (N=240)	
		Never true	184 (76.7)
		Sometimes true	45 (18.8)
		Often true	11 (4.6)
Transportation needs			
Have you not seen a doctor because you didn’t have a way to get to the clinic or hospital? (N=249)		In the past 12 months, has lack of transportation kept you from medical appointments or from getting medications? (N=246)	
Yes	20 (8.0)	Yes	18 (7.3)
		In the past 12 months, has lack of transportation kept you from meetings, work, or getting things needed for daily living?	
		Yes	19 (7.6)
Financial strain			
Did you feel there was not enough money for items like clothing or furniture?		How hard is it for you to pay for the very basics like food, housing, medical care, and heating? (N=249)	
Yes	85 (34.0)	Not hard at all	96 (38.6)
Have you been unemployed and looking for work? (N=249)		Not very hard	64 (25.7)
Yes	49 (19.7)	Somewhat hard	69 (27.7)
		Hard	11 (4.4)
		Very hard	9 (3.6)
Lack of social support			
Have problems getting childcare or elder care made it difficult for you to work or get to appointments? (N=245)		Marital status (N=246)	
Yes	12 (4.9)	Married	139 (56.5)
		Widowed	15 (6.1)
		Divorced	33 (13.4)
		Separated	12 (4.9)
		Never married	27 (11.0)
		Living with partner	20 (8.1)
		In a typical week, how many times do you talk on the phone to family, friends, or neighbors? (N=248)	
		Never	9 (3.6)
		Once a week	51 (20.6)
		Twice a week	35 (14.1)
		Three times a week	36 (14.5)
		More than 3 times a week	117 (47.2)
		How often do you get together with friends or relatives? (N=243)	
		Never	32 (13.2)
		Once a week	109 (44.9)
		Twice a week	43 (17.7)
		Three times a week	13 (5.3)
		More than 3 times a week	46 (18.9)
		How often do you attend church or religious services? (N=244)	
		Never	130 (53.3)
		1–4 times per year	28 (11.5)
		More than 4 times per year	86 (35.2)
		Do you belong to any clubs or organizations such as church groups, unions, fraternal or athletic groups, or school groups? (N=245)	
		Yes	89 (36.3)
		No	156 (63.7)
Additional questions			
Have you needed to see a doctor but could not because it costs too much? (N=248)		How often do you attend meetings of the clubs or organizations you belong to? (N=241)	
Yes	41 (16.5)	Never	155 (64.3)
		1–4 times per year	22 (9.1)
		More than 4 times per year	64 (26.6)
Did you not take medications to save money?		Stress means a situation in which a person feels tense, restless, nervous or anxious or is unable to sleep at night because his/her mind is troubled all the time. Do you feel this kind of stress these days?	
Yes	30 (12.0)	Not at all	46 (18.4)
		Only a little	61 (24.4)
		To some extent	71 (28.4)
		Somewhat or rather much	48 (19.2)
		Very much	24 (9.6)

^a^ Unless otherwise defined in the table.

### SDoH screening feasibility

Two hundred and eighty-six patients with CLD were approached for the study, and 250 were enrolled for an 87% participation rate. During the study, 50 consecutive patients were timed via the REDCap application. Completion of SINCERE took on average 38 seconds, with a minimum of 7 seconds and a maximum of 203 seconds. The EMR screener, on average, was completed in 190s, with a minimum of 66s and a maximum of 504s. Nearly all (95%) of participants answered 100% of the SINCERE, and 85% answered 100% of the EMR screener. When participants were compared by survey completeness, participants who answered 100% of the EMR and SINCERE questions versus those missing 1+ question, there was a statistically significant association found with Hispanic ethnicity, education level, annual income, living situation, and age (Supplemental Table S2, http://links.lww.com/HC9/C42).

### SDoH screening acceptability

Most respondents (69%) were comfortable or very comfortable completing the survey (including both SDoH screeners), and 52% felt it took “no effort at all.” Seventy-six percent stated the survey was acceptable or completely acceptable. Participants’ perception of the importance of SDoH screening and their relationship to health were overall positive; 59% agreed or strongly agreed that SDoH screeners “will help identify social factors impacting my health and liver disease.” Finally, 73% felt confident or very confident in their ability to complete SDoH screeners at each hepatology visit (Table 3).

**TABLE 3:** Participants’ reported acceptability of answering social drivers of health questions

	N (%)
How comfortable or uncomfortable did you feel while completing the survey? (N=244)	
Very uncomfortable	17 (7.0)
Uncomfortable	6 (2.5)
Neutral	53 (21.7)
Comfortable	82 (33.6)
Very comfortable	86 (35.2)
How much effort did it take to complete the survey? (N=247)	
No effort at all	129 (52.2)
A little effort	76 (30.8)
Moderate effort	33 (13.4)
A lot of effort	5 (2.0)
Huge effort	4 (1.6)
How fair or unfair is it for the clinic to ask you questions about the topics in this survey? (N=246)	
Very unfair	6 (2.4)
Unfair	2 (0.8)
Neutral	69 (28.0)
Fair	84 (34.1)
Very fair	85 (34.6)
To what extent do you agree or disagree that this survey improved your healthcare provider’s ability to identify social factors that may impact liver disease? (N=242)	
Strongly disagree	2 (0.8)
Disagree	8 (3.3)
Neutral	100 (41.3)
Agree	88 (36.4)
Strongly agree	44 (18.2)
To what extent do you agree or disagree with the following statement: It is clear to me how the questions in this survey will help identify social factors impacting my health and liver disease (N=241)	
Strongly disagree	5 (2.1)
Disagree	13 (5.4)
Neutral	82 (34.0)
Agree	94 (39.0)
Strongly agree	47 (19.5)
How confident or unconfident do you feel in your ability to complete a survey like this at each liver clinic visit? (N=245)	
Very unconfident	7 (2.9)
Unconfident	7 (2.9)
Neutral	52 (21.2)
Confident	94 (38.4)
Very confident	85 (34.7)
To what extent do you agree or disagree with the following statement: Completing the surveys interfered with my other priorities (N=246)	
Strongly disagree	77 (31.3)
Disagree	71 (28.9)
Neutral	65 (26.4)
Agree	26 (10.6)
Strongly agree	7 (2.8)
Overall, how acceptable or unacceptable was the survey to you? (N=245)	
Completely unacceptable	1 (0.4)
Unacceptable	1 (0.4)
Neutral	56 (22.9)
Acceptable	100 (40.8)
Completely acceptable	87 (35.5)

Among the 23 participants who selected uncomfortable or very uncomfortable, 70% (N=16) were women, 74% (N=17) were non-Hispanic White, 17% (N=4) were Hispanic or Latino/a, 4% (N=1) were Asian, and 4% (N=1) were Black or African American. Over one-third, 39%, completed a high school education or less, 26% were unemployed or disabled, 34% reported an annual income of <$35,000, and 86% live in an urban area. Proportions of birthplace (United States vs. outside United States), English as a second language, and language preference with providers were all comparable to the total study population.

### Factors associated with acceptability

The overall acceptability score of SDoH screening did not vary by gender identity, race or ethnicity, sexual orientation, employment, living situation, financial strain, geographic categorization, birthplace, or language. However, there was a significant association with level of education (*p*=0.015), as well as income (*p*=0.031): the acceptability scores were lower among participants with fewer years of education and lower annual income.

### Comparison of SDoH screening domains

A comparison of the results from EMR and SINCERE is shown in Table 4. Among each domain, the prevalence identified using each screener, the agreement between the screeners, and sensitivity, and specificity of SINCERE as compared to EMR are shown in the table.

**TABLE 4:** Social drivers of health prevalence and screener comparisons

	Value	95% CI
Food insecurity		
EMR prevalence	0.30	(0.24, 0.36)
SINCERE prevalence	0.26	(0.20, 0.32)
Cohen kappa	0.63	(0.52, 0.74)
McNemar	2.78	0.096 (*p* value)
Sensitivity	0.79	(0.69, 0.89)
Specificity	0.87	(0.82, 0.92)
Positive predictive value	0.68	(0.52, 0.79)
Negative predictive value	0.92	(0.88, 0.96)
Transportation needs		
EMR prevalence	0.09	(0.06, 0.13)
SINCERE prevalence	0.08	(0.04, 0.11)
Cohen kappa	0.43	(0.23, 0.63)
McNemar	0.73	0.394 (*p* value)
Sensitivity	0.53	(0.30, 0.75)
Specificity	0.94	(0.91, 0.97)
Positive predictive value	0.44	(0.23, 0.64)
Negative predictive value	0.96	(0.93, 0.99)
Financial strain		
EMR prevalence	0.08	(0.05, 0.12)
SINCERE prevalence	0.43	(0.37, 0.49)
Cohen kappa	0.17	(0.09, 0.26)
McNemar	82.18	<0.0001 (*p* value)
Sensitivity	0.17	(0.10, 0.24)
Specificity	0.99	(0.97, 1.00)
Positive predictive value	0.90	(0.77, 1.00)
Negative predictive value	0.61	(0.55, 0.68)
Housing instability		
PhenX prevalence	0.48	(0.41, 0.54)
SINCERE prevalence	0.24	(0.19, 0.30)
Cohen kappa	0.37	(0.27, 0.48)
McNemar	39.77	<0.0001 (*p* value)
Sensitivity	0.85	(0.76, 0.95)
Specificity	0.65	(0.57, 0.72)
Positive predictive value	0.43	(0.34, 0.53)
Negative predictive value	0.93	(0.88, 0.98)
Lack of social support		
EMR prevalence	0.04	(0.01, 0.07)
SINCERE prevalence	0.05	(0.02, 0.08)
Cohen kappa	0.06	(−0.13, 0.25)
McNemar	0.22	0.637 (*p* value)
Sensitivity	0.96	(0.94, 0.99)
Specificity	0.09	(0.00, 0.26)
Positive predictive value	0.96	(0.93, 0.98)
Negative predictive value	0.11	(0.00, 0.32)

Abbreviations: EMR, electronic medical record; SINCERE, Screener for Intensifying Community Referrals for Health.

Using EMR as the gold standard for food insecurity, SINCERE had a sensitivity of 79.0% and a specificity of 86.9%. Comparing the 2 screeners, a Cohen kappa of 0.63 shows substantial agreement, and a nonsignificant McNemar test (*p*=0.096) indicates there is not a statistically significant difference in the categorization of respondents between the 2 screeners.

Cohen kappa indicated slight agreement among the screeners for social support (0.06) and financial strain measures (0.17), fair agreement for housing instability (0.37), and moderate agreement for transportation needs (0.43). McNemar test for bias indicated there were not statistically significant differences in the categorization of respondents for the transportation (*p*=0.394) and social support measures (*p*=0.637), but there were significant differences for the categorization of respondents for the financial strain (*p*<0.0001) and housing measures (*p*<0.0001). Overall, these results look promising for food insecurity and transportation needs; okay for social support and housing; and poor for financial strain. Of note, the comparison for social support and transportation is limited by small sample size due to the low prevalence of each HRSN in our study population.

### Food insecurity

Thirty percent (N=72) of participants reported FI with the EMR screener, and 26% (N=62) with SINCERE. However, it was observed that, among specific groups, there was a clear difference in FI prevalence between the 2 screeners. Specifically, using the EMR screener, 46% (N=22) of Hispanic or Latino/a individuals were categorized as food insecure. On the other hand, SINCERE identified FI among only 31% of Hispanic participants. A similar difference was observed among AI/AN people, however, on a smaller scale due to sample size, 60% (N=9) as food insecure using EMR versus 47% (N=7) using SINCERE. Among non-Hispanic White participants, 24% had FI on both screeners.

### Transportation needs

Using the EMR screener, 9% (N=23) of participants reported having transportation needs, while 8% (N=19) had transportation needs based on SINCERE. The specificity of SINCERE compared to EMR was 94%; however, it had a lower sensitivity at 53%. Of note, SINCERE asks exclusively about healthcare-related transportation, described as the inability to get to “the clinic or hospital.”

### Financial strain

Based on SINCERE, 43% reported financial strain, compared to only 8% on the EMR screener. More specifically, 34% indicated there was not enough money for items like clothing or furniture, and 20% reported being unemployed within the past year and looking for work. Financial strain via the EMR screener was defined as responding with “hard” or “very hard” to paying for basics like food, housing, etc. If this definition is expanded to include the response “somewhat hard,” the prevalence of financial strain increases to 36%.

### Social support

Overall, the lack of social support was low, 5% via SINCERE and 4% via EMR, and agreement between the 2 screeners was high at 92%.

### Housing instability

For housing instability, SINCERE identified a prevalence of 24%, while prevalence using the PhenX housing screener was 48%. Overall agreement was low at 70%. However, PhenX covered housing instability and quality with questions about pests, mold, lack of heat, and water leaks, while SINCERE asked about the ability to afford mortgage, rent, and utilities.

## DISCUSSION

Among this study population of 250 patients with CLD receiving care in hepatology clinics, SDoH screening was feasible and acceptable, and SINCERE had acceptable convergent validity compared to the more well-established EMR screener. However, FI was reported by fewer participants using SINCERE, particularly among the Hispanic population. These data support the use of SDoH screening in hepatology clinics. Based on the goals of our healthcare system and our relationship with community partners, we propose the use of SINCERE to detect HRSN for our patients with CLD.^34^

There are numerous screeners available to assess for SDoH among patients.^20^ In general, any SDoH screener that (1) can be easily delivered in the healthcare setting; (2) patients will complete; and (3) helps identify modifiable SDoH, is an effective SDoH screener. Based on our findings, SINCERE was more feasible and acceptable. Moreover, it was developed in collaboration with local programs (Utah 2-1-1),^47^ which leads to direct action by community resources to address SDoH.^38^ Prior studies have successfully implemented SDoH screening with linkage to community resources, which led to improved clinical outcomes among select patient populations.^17^,^18^,^19^ Based on our findings, the modified SINCERE, or a similar screener, if conducted in hepatology clinics, could have a positive impact on patients with CLD.

The difference in FI prevalence between the 2 screeners is notable. Specifically, among Hispanic patients, FI prevalence was 15% lower using the single SINCERE FI question. Language may have played a role, although the sample size was small. Among the 10 participants who completed the screeners in Spanish, 4 had discordant responses—they reported FI on the EMR screener but not on SINCERE. Based on a prior SINCERE study, when given the option, Spanish-speaking participants were more likely to select “Decline to Answer” compared to English-speaking respondents.^48^ While language may account for some of the difference, in total, there were 7 Hispanic participants with discordant answers, and most (60%) of the participants who completed the screeners in Spanish reported the same for each screener, whether they had FI or not. In addition to language as an explanation, the option of “sometimes” as a response, which is available through the EMR screener but not SINCERE, may be less stigmatizing and therefore more likely to be selected than “yes.” These differences should be further explored through qualitative methods to better understand participants’ perceptions of the FI questions. Among the CLD population, the FI questions with the greatest sensitivity should be used as FI has been associated with higher risk of MASLD, MASLD-related advanced fibrosis, and all-cause mortality.^11^

SDoH screening acceptability was high overall; most participants were comfortable with the questions, considered screening to take little effort, and found the questions to be fair. Moreover, over half of the participants agreed that SDoH screening improves the ability of their providers to identify social factors that may impact their health and liver disease. Importantly, 73% were confident or very confident in their ability to complete SDoH screeners at each subsequent hepatology visit. On the other hand, participants with lower education or annual income considered SDoH screening less acceptable. These associations have been explored previously, and findings have varied among prior studies.^49^,^50^ One quantitative study conducted among nearly a thousand patients and caregivers across 9 states found that, although there were multiple factors associated with acceptability, the effects were small. They also found that prior exposure to SDoH screening, trust in clinicians, and recruitment from primary care (vs. the emergency department) and locations with higher prevalence of publicly insured or uninsured patients were all associated with higher screening acceptability.^20^ With standardized SDoH screening that occurs consistently in the healthcare setting, stigmatization may decrease. Additionally, if screening leads to action, such as connections to community resources, patients may better understand the purpose of screening and feel more comfortable sharing their needs and asking for help.

There are several limitations to this study. It was conducted at a single center that serves a predominantly non-Hispanic White population. However, our study population exhibits socioeconomic and geographic diversity, and we observed a high prevalence of social needs. Similarly, we used a screener that was developed at our institution, specific to our patient population and our local community resources, which limits its generalizability. However, this method may be replicated in or adapted to other healthcare systems, specifically to link SDoH screening to available resources. Second, it is a cross-sectional study that limits our ability to understand how repeat screening of SDoH may impact responses and acceptability. Nevertheless, for a comparison of 2 SDoH screeners, the cross-sectional study design is appropriate. Additionally, the comparison of the 2 screeners, specifically assessing the convergent validity of SINCERE, was limited by the use of very different measures for some of the domains. Third, acceptability was assessed for both screeners together; therefore, we could not determine if one screener was more acceptable than the other. Similarly, we did not compare all differing features of the 2 screeners, such as reading level. Although we consider this to be a strength of SINCERE, among this population, it cannot be known if this was an important difference. There are also limitations common to all survey-based studies, including participant self-report, specifically for SDoH, that can be stigmatizing, as well as recall and response bias. There were also notable strengths of the study, including the comparison of 2 SDoH screeners delivered to all participants in a randomly assigned order. We also had high participation and response rates. Moreover, all 250 participants were asked about the acceptability of SDoH screening in real-time.

In summary, there is a paucity of data on the feasibility and acceptability of SDoH screening among adults with CLD, despite the powerful impact SDoH has on CLD health disparities. Among this study population, we found that SDoH are prevalent, and screening in hepatology clinics is feasible and acceptable. These data support the use of SDoH screening in hepatology clinics. At our institution, where the detection of HRSN is the goal of SDoH screening, SINCERE was found to have acceptable convergent validity compared to EMR. Therefore, as a readily accessible, short, simple screener that captures HRSN to guide individualized care and referrals to available resources, it is an appropriate screening tool. Future longitudinal studies should assess SDoH screening in hepatology clinics on a larger scale and the connection to and utility of community resources.

## Supplementary Material
