# Supplementary material for: Feasible and acceptable social drivers of health screening among patients with chronic liver disease
Source: Hepatol Commun. 2025 Jun 30;9(7):e0758. doi: 10.1097/HC9.0000000000000758 (PMC12208639; doi:10.1097/HC9.0000000000000758)
Supplement: Supplementary file 1 [file hc9-9-e0758-s001.docx]

**Supplemental Table 1. Definitions of Responses for Positive and Negative Social Needs**

| **Survey** | **Construct** | **Positive/Yes** | **Negative/No** |
| --- | --- | --- | --- |
| PhenX | Housing instability | PhenX question “What is your living situation today” answered “I have a place to live today, but I am worried about losing it in the future” or “I do not have a steady place…” OR “Think about the place you live. Do you have problems…” answered with at least one check other than “None of the above” OR “Do you own or rent your home” answered as “Other arrangement…” OR “How often in the past 12 months would you say you were worried or stressed about having enough money to pay your rent/mortgage…” answered “Always,” “Usually,” “Sometimes,” or “Rarely” | PhenX question “What is your living situation today” answered “I have a place to live today” AND question “Think about the place you live. Do you have problems…” answered with “None of the above” AND question “Do you own or rent your home” answered as “Own” or “Rent” AND question “How often in the past 12 months would you say you were worried or stressed about having enough money to pay your rent/mortgage…” answered “Never” |
| SINCERE | Housing instability | At least one question answered “Yes” between the 3 Sincere questions “…not able to pay mortgage or rent,” “…not able to pay your utility bills,” “slept outside, in a shelter…” | All 3 of the following Sincere questions answered “No.” Questions: “…not able to pay mortgage or rent,” “…not able to pay your utility bills,” “slept outside, in a shelter…” |
| Electronic Medical Record (EMR) Screener | Food insecurity | One or both food security questions “….food would run out before you got money to buy more” and “….the food you bought just didn’t last and you didn’t have money to get more” answered “Often true” or “Sometimes true” | Both EMR food security questions answered “Never true.” Questions: “….food would run out before you got money to buy more” and “….the food you bought just didn’t last and you didn’t have money to get more” |
| SINCERE | Food insecurity | Sincere question “…not enough money for food” answered “Yes” | Sincere question “…not enough money for food” answered “No” |
| EMR | Transportation needs | At least one question answered “Yes” between EMR questions “..has lack of transportation kept you from medical appointments…” and “…has lack of transportation kept you from meetings, work…” | Both EMR transportation questions answered “No.” Questions: “..has lack of transportation kept you from medical appointments…” and “…has lack of transportation kept you from meetings, work…” |
| SINCERE | Transportation needs | Sincere question “have not seen a doctor because you didn’t have a way to get to the clinic…” answered “Yes” | Sincere question “have not seen a doctor because you didn’t have a way to get to the clinic…” answered “No” |
| EMR | Financial strain | EMR question “How hard is it for you to pay for the very basics like food, housing…” answered “Very hard” or “Hard” | EMR financial strain question answered “Somewhat hard” or “Not very hard” or “Not hard at all.” Question: ““How hard is it for you to pay for the very basics like food, housing…” |
| SINCERE | Financial strain | At least one question answered “Yes” between the Sincere questions “…not enough money for items like clothing or furniture” and “been unemployed and looking for work” | All of the following Sincere questions answered “No.” Questions: “…not enough money for items like clothing or furniture,” and “been unemployed and looking for work” |
| EMR | Lack of social support | Component 1: EMR question “Are you now married, widowed…” answered “Married” or “Living with partner” will score 1, and answered “Widowed,” “Divorced,” “Separated,” and “Never married” will score 0.  Component 2: Responses to the two questions “In a typical week how many times do you talk on the phone…” and “How often do you get together with friends or relatives” summed and multiplied to a yearly total, scores of >156 contacts/year will score 1, and scores of <156 contacts/year will score 0.  Component 3: EMR question “How often do you attend church…” answered “More than 4 times per year” will score 1, and answered “Never” or “1-4 times per year” will score 0.  Component 4: EMR question “Do you belong to any clubs or organizations…” answered “Yes” will score 1, and answered “No” will score 0.  The 4 component scores will be summed. Scores of 0 will be considered lack of social support. | Using the scoring system in cell to the left, scores of 2, 3, or 4. |
| SINCERE | Lack of social support | Sincere question “…problems getting child care or elder care” answered “Yes” | Sincere question 10 answered “No” |

**Supplemental Table 2. Completion Comparison of Social Drivers of Health Screeners**

|  | **Number of missing responses within EMR SDoH and Sincere surveys** | |  |
| --- | --- | --- | --- |
|  | **0 missing**  **“Completer”** | **1+ missing**  **“Non-Completer”** | **p-value** |
| **Totals** | 206 (82.4%) | 44 (17.6%) |  |
| **Race or ethnicity** |  |  |  |
| American Indian or Alaska Native | 13 (72.2%) | 5 (27.8%) | 0.331^1^ |
| Asian | 7 (87.5%) | 1 (12.5%) | 1.000^1^ |
| Black or African American | 5 (62.5%) | 3 (37.5%) | 0.153^1^ |
| Hispanic or Latino/a | 39 (72.2%) | 15 (27.8%) | 0.043^1^ |
| Middle Eastern or North African | - | - | - |
| Native Hawaiian or Pacific Islander | 3 (100.0%) | 0 (0.0%) | 1.000^1^ |
| White | 143 (85.6%) | 24 (14.4%) | 0.052^1^ |
| Missing | 2 | 0 |  |
| **Gender Identity** |  |  | 0.136^1^ |
| Man | 96 (88.1%) | 13 (11.9%) |  |
| Woman | 108 (78.8%) | 29 (21.2%) |  |
| Non-binary | 1 (100.0%) | 0 (0.0%) |  |
| Missing or “Prefer not to answer” | 1 | 2 |  |
| **Sexual Orientation** |  |  | 0.476^1^ |
| Straight | 178 (83.6%) | 35 (16.4%) |  |
| Gay, Bisexual, and “None of these describe me” | 14 (93.3%) | 1 (6.7%) |  |
| Missing or “Prefer not to answer” | 14 | 8 |  |
| **Highest level of school completed** |  |  | 0.001^1^ |
| Less than 12^th^ grade | 12 (60.0%) | 8 (40.0%) |  |
| High school graduate or GED | 41 (78.8%) | 11 (21.2%) |  |
| Some college, no degree | 53 (76.8%) | 16 (23.2%) |  |
| 2 year college degree/associate degree | 29 (93.5%) | 2 (6.5%) |  |
| 4 year college degree/bachelor’s degree | 42 (97.7%) | 1 (2.3%) |  |
| Graduate or professional degree | 27 (84.4%) | 5 (15.6%) |  |
| Missing | 2 | 1 |  |
| **Current occupational status** |  |  | 0.201^2^ |
| Employed | 86 (87.8%) | 12 (12.2%) |  |
| Unemployed for 1 year or more | 15 (78.9%) | 4 (21.1%) |  |
| Unemployed for 1 year or less | 10 (90.9%) | 1 (9.1%) |  |
| Homemaker | 9 (60.0%) | 6 (40.0%) |  |
| Student | 5 (100.0%) | 0 (0.0%) |  |
| Retired | 54 (80.6%) | 13 (19.4%) |  |
| Disabled | 18 (78.3%) | 5 (21.7%) |  |
| Other | 6 (100.0%) | 0 (0.0%) |  |
| Missing or “Prefer not to answer” | 3 | 3 |  |
| **Combined annual income** |  |  | 0.022^2^ |
| $0 - $9,999 | 17 (94.4%) | 1 (5.6%) |  |
| $10,000 - $14,999 | 6 (66.7%) | 3 (33.3%) |  |
| $15,000 - $19,999 | 8 (88.9%) | 1 (11.1%) |  |
| $20,000 - $34,999 | 19 (70.4%) | 8 (29.6%) |  |
| $35,000 - $49,999 | 12 (70.6%) | 5 (29.4%) |  |
| $50,000 - $74,999 | 33 (97.1%) | 1 (2.9%) |  |
| $75,000 - $99,999 | 28 (87.5%) | 4 (12.5%) |  |
| $100,000 - $199,999 | 46 (90.2%) | 5 (9.8%) |  |
| $200,000 or more | 16 (80.0%) | 4 (20.0%) |  |
| Missing or “Don’t know” or “Prefer not to answer” | 21 | 12 |  |
| **Living situation** |  |  | 0.009^1^ |
| I have a steady place to live | 197 (83.5%) | 39 (16.5%) |  |
| I have a place to live today, but I am worried about losing it in the future | 8 (80.0%) | 2 (20.0%) |  |
| I do not have a steady place to live | 0 (0.0%) | 2 (100.0%) |  |
| Missing | 1 | 1 |  |
| **Problems with living situation** |  |  |  |
| Pests such as bugs, ants, or mice | 16 (80.0%) | 4 (20.0%) | 0.752^1^ |
| Mold | 8 (80.0%) | 2 (20.0%) | 0.672^1^ |
| Lead paint or pipes | 1 (50.0%) | 1 (50.0%) | 0.304^1^ |
| Lack of heat | 2 (66.7%) | 1 (33.3%) | 0.420^1^ |
| Oven or stove not working | 2 (100.0%) | 0 (0.0%) | 1.000^1^ |
| Smoke detectors missing or not working | 7 (77.8%) | 2 (22.2%) | 0.646^1^ |
| Water leaks | 4 (100.0%) | 0 (0.0%) | 1.000^1^ |
| None of the above | 173 (84.4%) | 32 (15.6%) | 0.345^1^ |
| **Do you own or rent your home?** |  |  | 0.783^1^ |
| Own | 125 (83.3%) | 25 (16.7%) |  |
| Rent | 53 (85.5%) | 9 (14.5%) |  |
| Other arrangement | 13 (92.9%) | 1 (7.1%) |  |
| Missing or “Don’t know / Not sure” or “Decline to answer” | 15 | 9 |  |
| **How often in the past 12 months would you say you were worried or stressed about having enough money to pay your rent/mortgage?** |  |  | 0.670^1^ |
| Always | 6 (66.7%) | 3 (33.3%) |  |
| Usually | 13 (86.7%) | 2 (13.3%) |  |
| Sometimes | 36 (81.8%) | 8 (18.2%) |  |
| Rarely | 21 (84.0%) | 4 (16.0%) |  |
| Never | 105 (84.7%) | 19 (15.3%) |  |
| Missing or “Don’t know / Not sure” or “Not applicable” or “Decline to answer” | 25 | 8 |  |
| **Age** |  |  | <0.001^3^ |
| Mean (standard deviation) | 54.1 (14.2) | 63.0 (12.2) |  |
| Missing | 2 | 1 |  |
| **Birthplace** |  |  | 0.660^1^ |
| In the United States | 169 (82.8%) | 35 (17.2%) |  |
| Outside the United States | 33 (80.5%) | 8 (19.5%) |  |
| Missing | 4 | 1 |  |
| *If answered birthplace as “Outside the United States”*  **Years in the United States** |  |  | 0.285^3^ |
| Mean (standard deviation) | 33.5 (18.3) | 24.6 (8.9) |  |
| Missing | 0 | 1 |  |
| **First language** |  |  | 0.472^1^ |
| English | 172 (83.1%) | 35 (16.9%) |  |
| Spanish | 21 (75.0%) | 7 (25.0%) |  |
| Other: Arabic, Chinese, Danish, Danko, Greek, Haitian Creole, Korean, Mai Mai, Navajo, Russian, Sign Language, Vietnamese | 13 (86.7%) | 2 (13.3%) |  |
| **Geographic classification** |  |  | 0.211^1^ |
| Urban | 157 (80.9%) | 37 (19.1%) |  |
| Large rural city/town | 27 (87.1%) | 4 (12.9%) |  |
| Small and isolated small rural town | 21 (95.5%) | 1 (4.5%) |  |
| Missing | 1 | 2 |  |

1. Fisher Exact
2. Monte Carlo approximation of Exact Test
3. Wilcoxon Rank Sum
